# Cardiovascular Responses to Natural and Auditory Evoked Slow Waves Predict Post‐Sleep Cardiac Function

**DOI:** 10.1111/jsr.70354

**Published:** 2026-06-01

**Authors:** Giulia Alessandrelli, Stephanie Huwiler, Giulio Bernardi, Manuel Carro‐Domínguez, Fabia Stich, Rossella Sala, Florent Aziri, Anna Trippel, Susanne Markendorf, David Niederseer, Philipp Bohm, Pietro Cerveri, Francesca Siclari, Reto Huber, Nicole Wenderoth, Christian Schmied, Caroline Lustenberger

**Affiliations:** ^1^ Department of Electronics, Information and Bioengineering Politecnico di Milano Milan Italy; ^2^ Neural Control of Movement Lab, Institute of Human Movement Sciences and Sport, Department of Health Sciences and Technology ETH Zurich Zurich Switzerland; ^3^ MoMiLab IMT School for Advanced Studies Lucca Lucca Italy; ^4^ Department of Cardiology University Heart Center Zurich, University of Zurich Zurich Switzerland; ^5^ Department of Cardiology Hochgebirgsklinik Davos, Medicine Campus Davos Davos Switzerland; ^6^ Christine Kühne ‐ Center for Allergy Research and Education (CK‐CARE) Medicine Campus Davos Davos Switzerland; ^7^ Istituto Auxologico Italiano, IRCCS Milan Italy; ^8^ The Netherlands Institute for Neuroscience Amsterdam the Netherlands; ^9^ Centre Hospitalier Universitaire Vaudois Lausanne Switzerland; ^10^ The Sense Innovation and Research Center Lausanne and Sion Switzerland; ^11^ Center of Competence Sleep & Health Zurich University of Zurich Zurich Switzerland; ^12^ Neuroscience Center Zurich (ZNZ) University of Zurich, ETH Zurich Zurich Switzerland; ^13^ Child Development Centre University Children's Hospital, University of Zurich Zurich Switzerland; ^14^ Department of Child and Adolescent Psychiatry and Psychotherapy, Psychiatric Hospital Zurich University of Zurich Zurich Switzerland; ^15^ Future Health Technologies Singapore‐ETH Center, Campus for Research Excellence and Technological Enterprise (CREATE) Singapore Singapore

**Keywords:** auditory stimulation, blood pressure, cardiac function, heart rate, slow wave type

## Abstract

The importance of sleep as a key component of cardiovascular health has been increasingly recognized. Our previous research demonstrated that auditory‐enhanced slow waves increase cardiac function, but the underlying mechanisms behind these beneficial effects remain uncertain. In this study, we examined the influence of two types of slow waves on cardiovascular functions. 18 healthy middle‐aged men participated in a three‐night sleep lab experiment under three conditions: two auditory slow wave enhancement conditions and one SHAM control. We found that the strength of heart rate and blood pressure responses occurring concurrently with slow waves predicts post‐sleep cardiac function in men as measured through transthoracic echocardiography. Furthermore, we observed that highly synchronized type I slow waves, compared to lower‐amplitude type II slow waves, are primarily associated with increased cardiovascular responses (transient increase in heart rate and blood pressure). While auditory stimulation enhances both types of slow waves, they display distinct temporal dynamics, suggesting different underlying brain and body microstates. These distinct slow wave types may affect cardiovascular function differently, indicating that targeted slow wave stimulation could be a strategic approach to enhancing cardiac function.

**Trial Registration:** This study was registered under clinicaltrials.gov (NCT04166916)

## 1 Introduction

With humans spending approximately one‐third of their lives sleeping, sleep's implications impact the entire spectrum of health and disease. The bidirectional relationship between sleep and cardiovascular function has been increasingly recognized, with changes in sleep macro‐architecture being both a consequence and a contributor to cardiovascular diseases (Valenzuela et al. 2021; Kohansieh and Makaryus 2015; Cappuccio et al. 2011; Grandner et al. 2010).

Essentially the deepest stages of non‐rapid eye movement (NREM) sleep seem to be crucial for cardiovascular recovery and health. Specifically, slow waves, the hallmark oscillations of deep NREM sleep, may play a critical role in promoting cardiovascular function during sleep. Auditory stimulation during NREM sleep is a feasible approach to effectively modulate sleep slow waves (Lustenberger et al. 2022; Ngo et al. 2013; Ngo et al. 2015; Besedovsky et al. 2017; Grimaldi et al. 2019). However, the influence of auditory stimulation beyond modulating cortical activity, such as its impact on the autonomic nervous system, which could influence cardiovascular function during sleep, remains a topic of ongoing investigation (Grimaldi et al. 2019; Huwiler et al. 2023; Diep et al. 2022; Huwiler et al. 2022). Our group's recent findings demonstrated that auditory stimulation enhances slow waves and induces a cardiovascular response, including a transient increase in blood pressure and a biphasic heart rate change, while also increasing post‐sleep left ventricular function compared to a control condition (Huwiler et al. 2023). However, the underlying mechanisms linked to such cardiovascular benefits remain unclear.

There is growing evidence that two types of slow waves exist, which can be differentiated based on wave morphology, timing, and synchronization processes (Bernardi et al. 2018; Siclari et al. 2014). Type I slow waves, which exhibit a broad cortical synchronization, are predominantly observed during the early stages of sleep onset and likely result from subcortico‐cortical synchronization processes of neurons (Bernardi et al. 2018; Siclari et al. 2014). In contrast, type II slow waves, resulting from cortico‐cortical synchronization, become more prominent during the later stages of sleep onset (Bernardi et al. 2018; Siclari et al. 2014). K‐complexes, which represent the largest deflection observed in EEG readings during NREM sleep and can be triggered by external stimuli or occur spontaneously during sleep, are likely characterized by their substantial neuronal synchronization, potentially via subcortico‐cortical synchronization processes, and could thus be considered a specialized subtype of type I slow waves. They have been proposed to play an essential role in promoting cardiovascular homeostasis (de Zambotti et al. 2016) and were linked to a dynamic blood pressure and heart rate response, potentially through a short burst of sympathetic activity concurrent with their occurrence (de Zambotti et al. 2016; Greenlund et al. 2021; Sforza et al. 2000; Tank et al. 2003; Monstad and Guilleminault 1999; Somers et al. 1993). Given the potential different mechanisms underlying the occurrence of type I and type II slow waves, elucidating whether they display distinct influences on the cardiovascular system is crucial in understanding their implications in promoting cardiovascular health.

We determined whether these two types of slow waves are associated with distinct changes in cardiovascular activity during sleep. We provide insights into the relationship between the cardiovascular activation occurring with auditory slow wave enhancement and post‐sleep cardiac function. Moreover, we show that previously proposed distinctive types of slow waves based on cortical features are also occurring alongside distinctive cardiovascular dynamics, with type I slow waves evoking stronger cardiovascular responses compared to type II slow waves. Finally, we demonstrate that auditory stimulation differently evokes type I or type II slow waves during times of stimulation, leading to important insights on how auditory stimulation may result in cardiac benefits the next morning.

## 2 Methods

### 2.1 Participants and Experimental Procedure

Overall, 26 healthy male participants were enrolled in the randomized‐controlled trial (registered at ClinicalTrials.gov (NCT04166916), see Figure S1 for an overview of trial profile). The here presented analysis is a secondary analysis of the main results of this clinical trial, which were reported previously (Huwiler et al. 2023). Participants were recruited from the local community using advertisements on different platforms. All participants were of good general health, non‐smokers, reported a regular sleep–wake rhythm, had a body mass index between 17–30, and none of the participants had any cardiovascular disorder, which has been confirmed by an expert cardiologist. Furthermore, all participants had no psychiatric or neurological disease and no presence or suspicion of any sleep disorders. Exclusion criteria further encompassed any participant using on‐label sleep medication or medication impacting cardiovascular function, as well as those demonstrating low sleep quality during the screening night (defined as sleep efficiency below 75%; dropout rates and reasons are provided in Figure S1). Finally, 18 participants underwent the full three nights auditory stimulation protocol. All included participants provided written informed consent prior to the study and were compensated for their participation. The study was conducted following the declaration of Helsinki and approved by the cantonal Ethics Committee Zurich (BASEC2019‐01538).

The study design is illustrated in Figure 1. All participants underwent a screening period including a phone screening and a screening night to familiarize participants with the sleep lab environment and all measurement devices. To ensure hearing capabilities of 40 dB, we conducted a short audiometry test. After participants had passed the screening night, they were screened by an expert cardiologist for any cardiovascular comorbidities at the University Hospital Zurich before the first experimental night. Participants were asked to adhere to a regular sleep rhythm one week before the start of the experimental night (sleep duration of 7–9 h, bedtime *±*1 h to the scheduled bedtime in the sleep lab, no naps). Three nights before the main experimental night, participants had to sleep for 7.5 h *±* 0.5 h, with a bedtime *±*0.5 h to the scheduled bedtime. Adherence was verified through actigraphy (GENEActiv, Activinsights, Kimbolton, UK) and a sleep diary.

**FIGURE 1:**
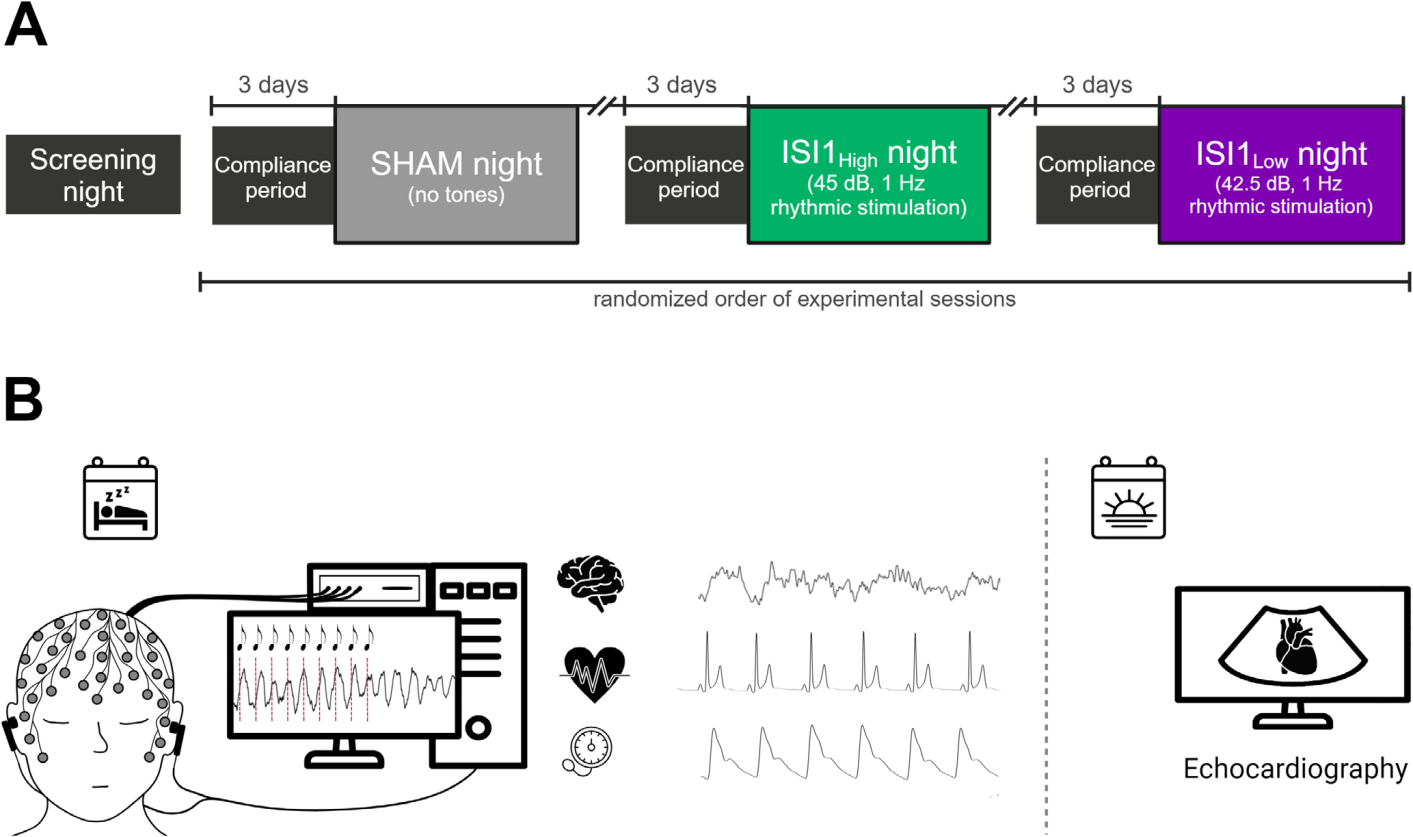
Study protocol of clinical trial. (A) First, there was a screening period where the eligibility of participants was checked by an initial phone screening followed by a screening night in the sleep laboratory. If all inclusion/exclusion criteria were fulfilled, participants were invited to a cardiovascular screening to verify the absence of cardiovascular diseases. Afterwards, the experimental phase including three experimental nights featuring one of three conditions (no auditory stimulation (SHAM), slightly lower volume auditory stimulation (*ISI1*_Low_), or slightly higher volume (*ISI1*_High_)) took place. Three days before each experimental night, a compliance period started where participants had to adhere to a regular sleep rhythm. The intervention condition was administered in a randomized order. (B) Overview of here reported measurements during the experimental night. Participants got a 7.5 h sleep opportunity where polysomnography, electrocardiography, and continuous blood pressure were recorded. During sleep, one of the three stimulation conditions according to randomization protocol was applied through an electroencephalography (EEG)‐feedback system. In the morning, at the end of each experimental night, an echocardiography recording was obtained by an expert cardiologist.

On the day of each experimental night, participants had to abstain from naps, alcohol, intensive sports, caffeine, and food 5 h before arriving at the sleep lab. We attached a high‐density EEG system (Geodesic Sensor Net, Magstim EGI, Eugene, USA), and two ECG electrodes (Gold Genuine Grass electrodes, Natus Medical Inc., Pleasanton, US) under the right collar bone and on the left rib cage, respectively. All impedances of EEG and ECG electrodes were kept below 40 k ohm. Afterward, a continuous non‐invasive blood pressure monitoring system (Finapres Novascope, Finapres Medical Systems BV, Enschede, Netherlands) was attached and calibrated to ensure proper attachment and signal quality. After lights out, participants could sleep for 7.5 h while polysomnography and ECG were recorded (BrainProducts GmbH, Gliching, Germany) at a sampling rate of 500 Hz. Beat‐to‐beat blood pressure was continuously measured with a sampling rate of 200 Hz. After a constant morning routine including additional tests (including autonomic manoeuvres, and cognitive and behavioural testing, not reported here), participants went to the University Hospital Zurich for a transthoracic echocardiography (TTE).

### 2.2 Auditory Slow Wave Stimulation Protocol

To investigate the effects of slow waves on cardiovascular function, participants completed a randomized crossover experimental protocol with three stimulation nights. We applied two stimulation conditions that were selected based on their dynamic effects on the brain and cardiovascular system (Huwiler et al. 2023) and compared them to a SHAM (no stimulation) condition. Namely, we used 1 Hz rhythmic EEG feedback‐controlled auditory stimulation of a 50 ms burst of pink noise with a sound level of 45 dB (*ISI*1_High_) and a sound level of 42.5 dB (*ISI*1_Low_). We used two different sound volumes to achieve a dose‐dependency effect and to investigate which dose is still effective in inducing possible cardiovascular responses. Auditory stimulation was delivered using a real‐time system programmed in OpenViBE (Renard et al. 2010). The auditory stimulation logic included the detection of stable NREM sleep based on EEG power in three different frequency bands (low delta: 2–4 Hz, high delta (3–5 Hz), and high beta (20–30 Hz)). During the first 10 min of uninterrupted detected NREM sleep, an individual beta power threshold was calculated. Furthermore, we calculated a correlation index between the two EOG electrodes, serving to minimize stimulations during high anti‐correlation periods, likely indicating non‐NREM sleep periods. In addition, absolute EEG signal amplitude served to detect movements. Stimulation was enabled whenever stable NREM sleep was detected (e.g., after 10 min of detecting uninterrupted NREM sleep for the first time, and after 3 min for every subsequent NREM episode), beta power was below the individual threshold, low anti‐correlation EOG index, no detected movement, and delta power exceeding a predefined threshold. Finally, the first stimulation of a stimulation window could occur whenever a first‐order phase‐locked loop (Ferster et al. 2019) reached the target phase of 50° (up‐phase stimulation). The remaining 9 stimulations during the ten‐second ON window were presented with a 1 Hz rhythm (inter‐tone interval of 1 s), independent of the underlying phase of ongoing slow waves. Stimulations were presented through Etymotic insert earphones (Etymotic Research Inc., ER 3C). The ten‐second ON (auditory stimulation presented) was followed by a ten‐second OFF (no auditory stimulation presented) window.

### 2.3 EEG Data Processing and Slow Wave Classification

Data processing was conducted in MATLAB (R2022b, MathWorks Inc., Natick, MA), with the initial EEG recording, sampled at 500 Hz, being down‐sampled to 200 Hz. Employing the EEGLAB toolbox (Delorme and Makeig 2004), pre‐processing involved detrending to remove slow drifts and low‐frequency artefacts, using a 1.5‐s sliding window with a 0.02 s step. The PREP pipeline algorithm (Bigdely‐Shamlo et al. 2015) was used to identify poor‐quality EEG channels through amplitude assessment, signal‐to‐noise ratio estimation, inter‐channel correlation analysis, and RANSAC‐based channel prediction. Identified noisy channels were temporarily excluded to prevent data distortion. Further, we applied a 50 Hz notch filter and a 0.5–45 Hz Butterworth bandpass filter using TESA within EEGLAB (Rogasch et al. 2017). Independent Component Analysis was then conducted to decompose EEG recordings, isolating neural from non‐neural signals (Delorme et al. 2007; Makeig et al. 2004; Makeig et al. 1995). Components were classified using the *ICLabel* algorithm (Pion‐Tonachini et al. 2019), distinguishing between brain‐related and non‐brain‐related activities. Components with a “brain” classification probability lower than 25% were rejected. Among the remaining components, we retained both ICs classified as “brain” activity and ICs labelled as “non‐brain” when the probability ratio between “brain” and the first “non‐brain” component was greater than 0.8. All rejected components were additionally verified through systematic manual inspection based on established criteria including scalp topography, time course, and spectral features (Grollero et al., 2025). Finally, previously excluded noisy channels were reintegrated using spherical spline interpolation and the data was re‐referenced to mastoid electrodes for further analysis.

An automated, two‐step algorithm was employed to identify slow waves in EEG recordings (Bernardi et al. 2018). The first step involved reducing multi‐channel EEG data to a single‐channel representation. For each time point, we selected and averaged the three most negative amplitude values across all available channels after excluding the most negative extreme values and data from peripherally situated electrodes. This procedure allowed us to obtain a composite signal representing the negative envelope of the EEG signals across the considered set of electrodes. A Butterworth band‐pass filter with a frequency range of 0.5 to 40 Hz was applied to obtain both zero‐mean signal centering and noise reduction. Potential slow waves were then selected using a duration‐based criterion applied to detected negative half‐waves. Specifically, a waveform was classified as a slow wave if its negative component had a duration between 0.25 and 1 s and it was observed during the NREM 2 or NREM 3 sleep stages. A representative figure depicting the two waveforms is provided in Figure S2.

Following the identification of slow waves in EEG recordings, we conducted a detailed examination of individual slow wave events, extracting their morphological and dynamic features. These features included the negative slope (*μ*V*/*s), calculated as the gradient between the wave's onset and its maximum negative peak, reflecting the rate of neuronal down‐state entry (Bernardi and Siclari 2019; Steriade et al. 2001). The positive slope (*μ*V*/*s), computed from the wave's maximum negative peak to the point where the waveform becomes positive, quantified the transition into an up‐state (Bernardi and Siclari 2019; Steriade et al. 2001). Additionally, an involvement score (dimensionless) was determined, representing the percentage of channels contributing to the slow wave, calculated around the maximum negative peak with a fixed amplitude threshold (–5 *μ*V).

These features led to the definition of the synchronization score (mV*/*s), a key metric for distinguishing slow wave types based on cortical involvement and synchronization speed (Bernardi and Siclari 2019). The synchronization score was instrumental in classifying slow waves into type I and type II categories, based on dynamic thresholds derived from the synchronization score distribution in each EEG recording. After calculating the synchronization scores for each EEG recording, two critical thresholds were determined to categorize slow waves. These thresholds were calculated by adding the median value of the synchronization scores to its median absolute error, multiplied by a specific numerical factor. A factor of 3 defined the lower threshold, while a factor of 4 established the upper threshold. Consequently, slow waves with synchronization scores below the lower threshold were classified as Type II, and those above the upper threshold as Type I. Slow waves falling between these two thresholds were marked as indeterminate and excluded from analysis when focusing on the differences between types I and II.

(1) $$\begin{array}{c} \text{synchronization score}=\text{involvement score}\\ \;\times \frac{\text{positive slope}+\text{negative slope}}{2}\times \frac{1}{1000} \end{array}$$

Finally, an additional classification layer was implemented to distinguish stimulated slow waves from spontaneous ones, depending on whether they coincided with the ON period of the stimulation windows. It is crucial to note that during the SHAM condition, stimulated slow waves are spontaneous slow waves occurring within the ON windows, which are automatically set through the EEG‐feedback algorithm, serving as a control comparison.

### 2.4 Heart Rate and Blood Pressure During Sleep

Continuous blood pressure and heart rate are derived 2 s before to 18 s after each slow wave onset, respectively from the continuous non‐invasive blood pressure monitoring system and the electrocardiogram. These data, extracted within the stimulation windows, were reported here (Huwiler et al. 2023). Please note that, unlike in the previous publication (Huwiler et al. 2023), we analyzed the cardiovascular response for each slow wave within the stimulation window (with time 0 s representing the onset of a slow wave) rather than aligning it with the start of the stimulation window. Briefly, electrocardiogram R‐waves were detected automatically and visually corrected if necessary using PhysioZoo (Behar et al. 2018). Segments of non‐detectable R‐waves due to poor data quality were excluded. The generated RR interval sequences were further processed with MATLAB. IHR was calculated by dividing 60 through the specific RR interval and interpolating the beat‐to‐beat data to 200 Hz by using linear interpolation. Segments of bad quality or artefacts of nocturnal diastolic and systolic blood pressure were removed based on detected heart rate outliers, using the function *isoutlier* with the option *gesd* in MATLAB. We further removed segments with less than three valid data points within ten seconds, as the low number of valid measurements could indicate bad recording quality. Blood pressure data points were interpolated to 200 Hz by using linear interpolation.

### 2.5 Echocardiography

Each morning following the experimental night, an experienced cardiologist performed a TTE on a GE Healthcare E95 echocardiography station (GE Healthcare Systems, Wisconsin, USA). The following measurements were collected: left‐ventricular end‐diastolic diameter (LVEDD), left‐ventricular end‐diastolic volume (LVEDV), left ventricular end‐systolic volume (LVESV), LVEF, left‐ventricular longitudinal strain (strain), left‐ventricular tissue doppler (E‐wave, A‐wave, E/A ratio, lateral e’ wave, E/e’ ratio), trans‐tricuspidal systolic right ventricular gradient, right ventricular end‐diastolic area (RVareaD), and TAPSE. The findings related to auditory stimulation conditions were partially detailed here (Huwiler et al. 2023).

### 2.6 Statistical Analysis

Statistical analyses were conducted in Python (version 3.9.13), MATLAB (version R2022b), and R (R Core Team 2013) (R Studio version 1.2.5033). Because of a technical failure in acquiring one participant's overnight EEG data of the SHAM night, this participant was completely excluded from all analyses, resulting in 17 subjects included in the here presented analyses (all males, aged 45.5 ± 9.86 years). We excluded two additional participants for the analyses leading to Figures 2 and 3 because their ECG signal was too compromised (non‐detectable R peaks) during the periods of detected type I and II slow waves. Therefore, 15 participants were included in these final analysis. In Figure 4, we had to exclude another participant, as the low incidence of type I stimulated slow waves in combination with a noisy ECG signal during the time of type I slow wave occurrence led to low trial numbers. Therefore, only 14 subjects were included in this plot.

**FIGURE 2:**
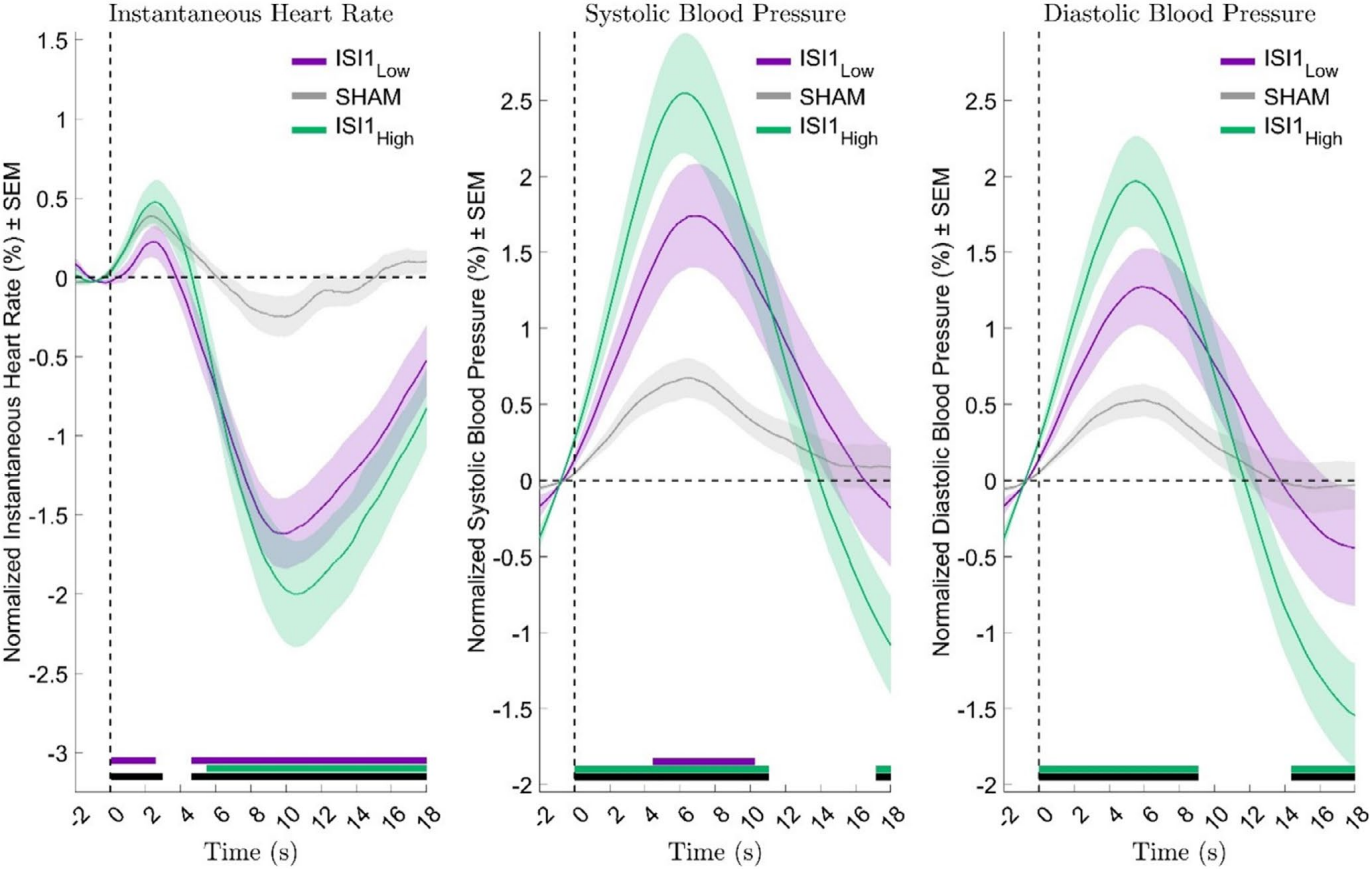
Cardiovascular responses of detected slow waves occurring during stimulation windows. Auditory stimulation significantly affects diastolic and systolic blood pressure and instantaneous heart rate within the three conditions (*ISI1*_High_, *ISI1*_Low_, and SHAM (no tones applied)), as indicated through the black bars. Here, all detected slow waves occurring during stimulation ON windows were included. The cardiovascular data are normalized against the 2‐s before the slow wave onset, including 15 for heart rate and 17 subjects for diastolic and systolic blood pressure over three experimental night conditions. Timepoint 0 represents the start of the slow waves. Linear mixed‐effects models were employed including subject as a random factor and condition as a fixed factor for each time point, and corrected for multiple comparisons using the false discovery rate Benjamini‐Hochberg correction. Statistically significant differences between conditions (*p* < 0.05) are represented by black lines. Post hoc *p*‐values were further corrected for multiple comparisons using the Bonferroni correction, resulting in a corrected *p*‐value threshold of 0.025. Significant post hoc comparisons between auditory stimulation conditions and the SHAM control are indicated by purple lines for *ISI1*_Low_ and green lines for *ISI1*_High_.

**FIGURE 3:**
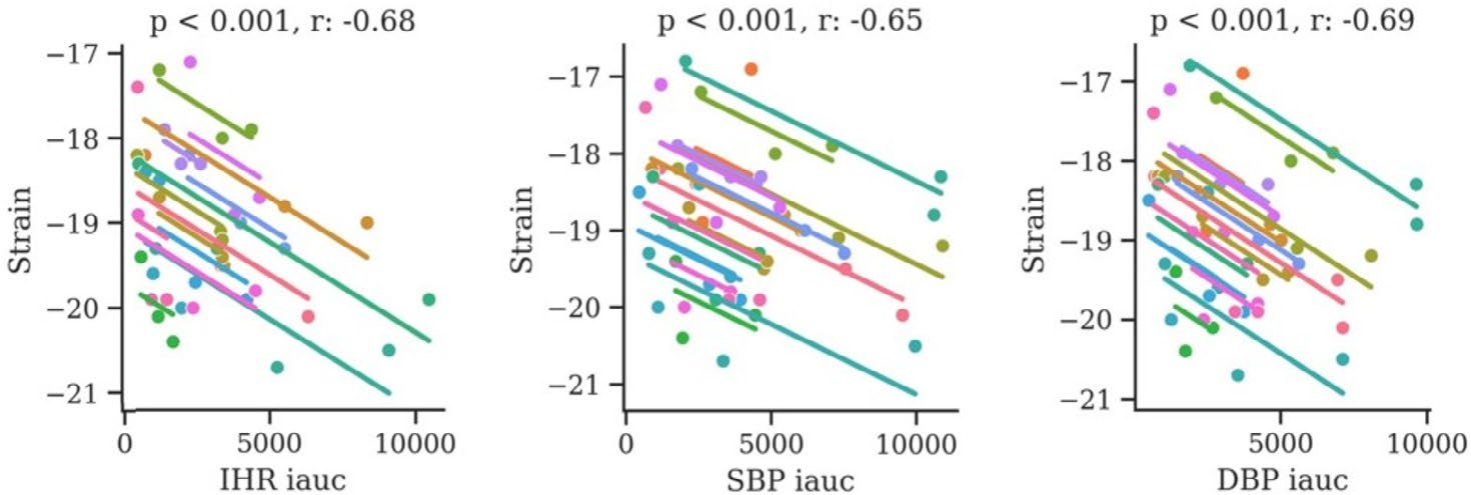
Within‐subject relationship of post‐sleep global longitudinal strain with cardiovascular responses to detected slow waves in auditory stimulation windows. Data is incorporated for 15 subjects for instantaneous heart rate (IHR) and 17 subjects for diastolic and systolic blood pressure (DBP, SBP) using repeated measures correlations. Cardiovascular metrics were computed as integrated area under the curve (iAUC) and quantified in a time frame from 2 s before to 18 s after each slow wave onset occurring in a simulation ON window. Repeated measures linear correlations were calculated to associate transthoracic echocardiography parameters with the iAUC of the averaged cardiovascular responses from auditory‐stimulated slow waves in each sleep recording.

**FIGURE 4:**
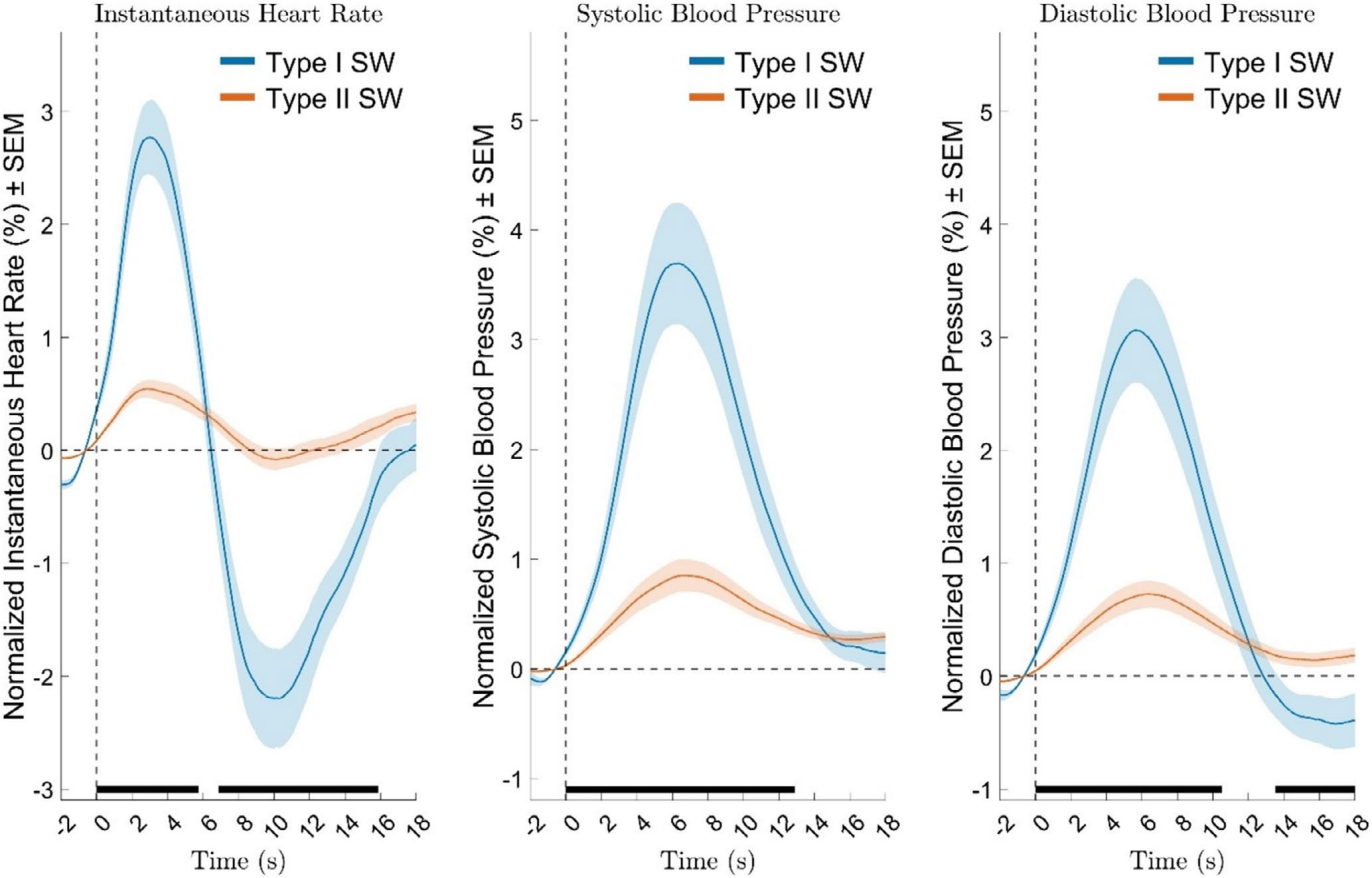
Distinct cardiovascular responses to type I and II slow waves in unstimulated sleep. Type I (including K‐complexes) and type II slow waves are accompanied by different cardiovascular responses, as evidenced in the data from 17 subjects for blood pressure and 14 for instantaneous heart rate across the unstimulated (SHAM) night. All slow waves independent of any stimulation window were included in this analysis. The cardiovascular data are normalized against the 2‐s baseline before the slow wave onset. Stronger reactions in instantaneous heart rate, diastolic, and systolic blood pressure coincide after the onset (Time: 0 s) of type I slow waves, whereas type II slow waves reveal smaller responses. Linear mixed‐effects models were employed including subjects as a random factor and type of slow wave as a fixed factor for each time point, and corrected for multiple comparisons using the false discovery rate Benjamini‐Hochberg correction. Statistically significant differences between type of slow wave (*p* < 0.05) are indicated by black lines.

To compare post‐sleep TTE parameters between conditions that have not been reported in (Huwiler et al. 2023), we used linear mixed‐effects models with fixed factors condition and random factor subject using the software package lme (Bates et al. 2015) in R. Here, if the model was significant for the condition, we derived post hoc *p*‐values for both auditory stimulation conditions compared to SHAM using paired *t*‐tests and correcting for multiple comparisons applying the Bonferroni correction. Using Python, we computed the repeated measures linear correlation (Vallat 2018) to correlate TTE parameters with cardiovascular responses from auditory‐stimulated slow waves. For Figures 2, 3, 4, 5, we performed linear mixed‐effects models using the MATLAB function *fitmod*, entering the subject as a random factor and the condition as a fixed factor (*α* = 0.05) for every timepoint. Given the repeated measures analysis for each time point, we applied the false discovery rate Benjamini‐Hochberg correction for multiple comparisons. Post hoc *p*‐values were obtained through paired *t*‐tests between *ISI1*_High_ and SHAM, and *ISI1*_Low_ and SHAM and corrected for multiple comparisons between conditions by using the Bonferroni‐corrected *p*‐value threshold of 0.025. Otherwise, *p* < 0.05 was considered as significant.

**FIGURE 5:**
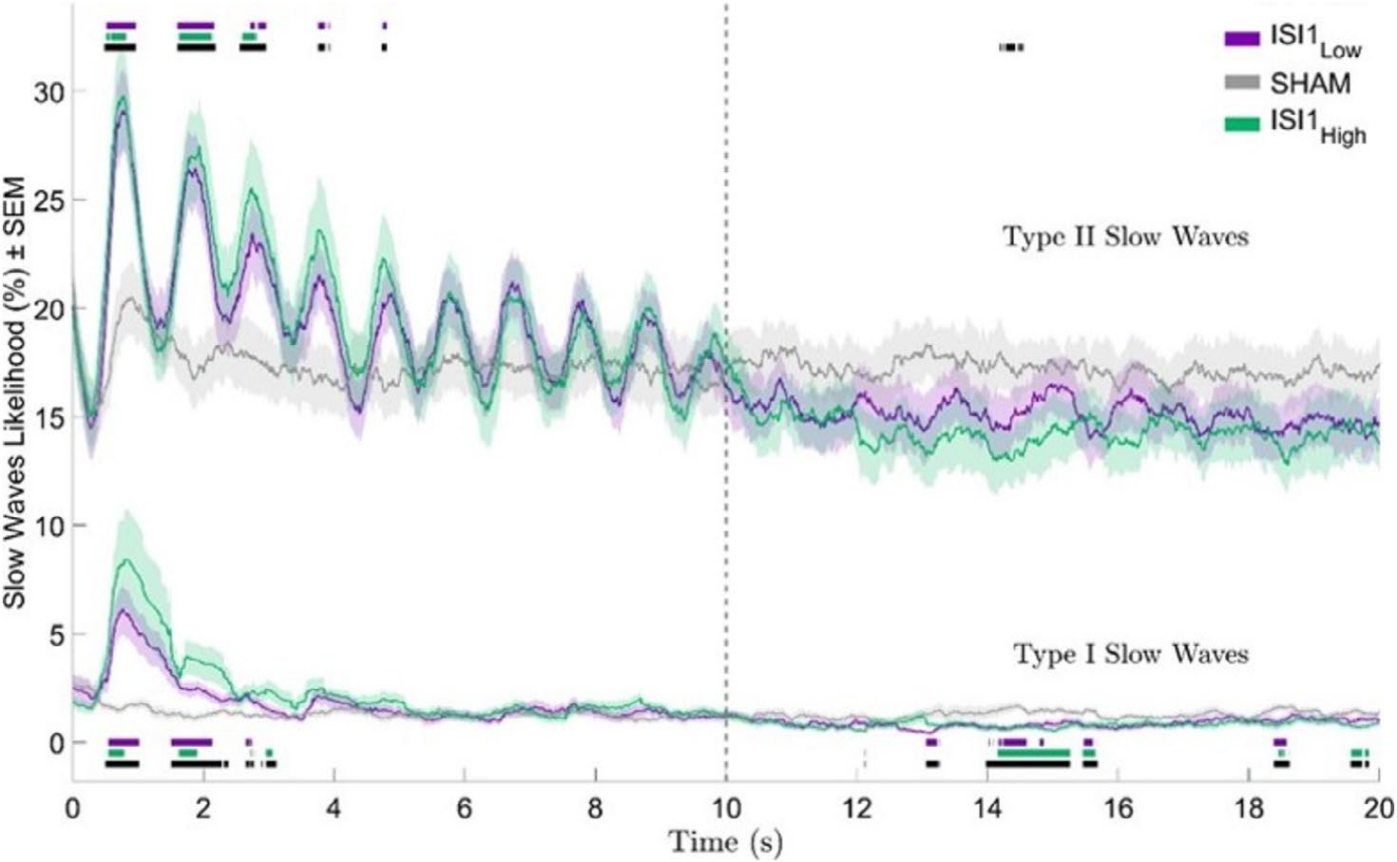
Different impact of auditory stimulation on type I and type II slow waves. Auditory stimulation differentially affects type I (including K‐complexes) and type II slow waves occurrences during stimulation ON and OFF window, based on data from 17 subjects over three experimental night conditions. Type II slow waves show an increase in likelihood during ON window stimulation each second, followed by a rapid decrease, a trend consistent across different volume levels. In contrast, type I slow waves (including K‐complexes), are characterized by a pronounced initial response. To assess the statistical significance, linear mixed‐effects models were employed, including subjects as a random factor and conditions as a fixed factor for each time point, and corrected for multiple comparisons using the false discovery rate Benjamini‐Hochberg correction. Differences between conditions that reached statistical significance (*p* < 0.05) are denoted by black lines. For post hoc comparisons, *p*‐values were adjusted for multiple testing using the Bonferroni correction, establishing a new threshold of 0.025. Significant post hoc comparisons between the auditory stimulation conditions and the SHAM control are marked with purple lines for *ISI1*_Low_ and green lines for *ISI1*_High_.

## 3 Results

### 3.1 Auditory Slow Wave Stimulation Induces Dynamic Heart Rate and Blood Pressure Response

To understand the link between auditory enhanced slow waves and cardiovascular dynamics during sleep, we here extracted heart rate and blood pressure from the 2 s before to 18 s after detected slow wave onsets occurring during stimulation ON windows. Afterward, we normalized each signal against its average value in the 2‐s baseline period. We then calculated the average of these normalized responses for each experimental condition (*ISI*1_High_, *ISI*1_Low_, and SHAM) within individual subjects and all participants. As seen in Figure 2, there is a significant difference (*p* < 0.05) across experimental conditions, indicating altered cardiovascular responses in both auditory stimulation conditions relative to the SHAM control. Auditory stimulation induced a stronger heart rate response, particularly after 6 s of detected slow wave onset, where the heart rate was lowered more strongly. Furthermore, blood pressure showed a temporal increase in stimulation conditions compared to SHAM control.

### 3.2 Slow Wave Associated Cardiovascular Responses Predict Post‐Sleep Cardiac Functions

We then established how these induced cardiovascular responses during sleep slow waves predict post‐sleep left‐ventricular systolic and diastolic function changes that we have previously reported in (Huwiler et al. 2023). Therefore, we investigated how the strength of the induced cardiovascular responses is related to post‐sleep cardiac parameters. We extracted the cardiovascular responses within a window from 2 s before to 18 s after the detected onset of both types of slow waves that occurred during marked stimulation windows (stimulation ON window) of all conditions (note that for the SHAM night, slow waves were only detected but not stimulated). The average of each cardiovascular response for every subject across each night was computed, followed by calculating the integrated area under the curve (iAUC) to ensure a positive valuation of each area under the response curve. Afterwards, we used repeated measures correlations between the iAUC cardiovascular metrics (instantaneous heart rate (IHR), systolic blood pressure (SBP), and diastolic blood pressure (DBP)) during sleep and post‐sleep TTE‐derived parameters that reflect cardiac function (global longitudinal strain, left‐ventricular ejection fraction (LVEF), ratio of early diastolic filling to annular velocity (E/e’), and tricuspid annular plane systolic excursion (TAPSE)). Because we did not measure BP directly before the TTE measurement, we compared a BP measurement around 1 h after waking up between conditions using a linear mixed‐effect model approach. We found no significant difference (F = 0.68 (Kohansieh and Makaryus 2015; Bates et al. 2015), *p* = 0.52, see Figure S3) between the conditions.

As illustrated in Figure 3, we found a strong negative repeated measures correlation between the strength of IHR, SBP, DBP, and global longitudinal strain. Global longitudinal strain maps the complex contraction of the left‐ventricular myocardium, predominately of the subendocardial longitudinally oriented fibres (Sugimoto et al. 2017), and is an established marker assessing left‐ventricular systolic function (Bergler‐Klein 2015; McDonagh et al. 2021). Our results indicate increased left‐ventricular systolic function (evidenced by a more negative global longitudinal strain) with an increase in cardiovascular response. Furthermore, we found strong positive repeated measures correlations between the cardiovascular responses and LVEF, the current standard clinical method to assess left‐ventricular systolic function (see Table 1) (Klaeboe and Edvardsen 2019). E/e’ was negatively correlated with the peak‐to‐peak IHR and blood pressure response (see Table 1), indicating increased left‐ventricular diastolic function (Nagueh et al. 2016). Therefore, pointing towards increased relaxation of the left cardiac ventricle relating to increased cardiovascular response. Lastly, we investigated whether the cardiovascular iAUC metrics influence right‐ventricular systolic function and, therefore, the contraction of the right ventricle (TAPSE). This cardiac metric was not reported previously in (Huwiler et al. 2023). Using a linear mixed effects model approach, we found stimulation conditions to significantly influence post‐sleep TAPSE (F = 4.7 (Kohansieh and Makaryus 2015; Bates et al. 2015), *p* = 0.016, see Figure S4), and post hoc comparisons using paired *t*‐tests revealed a significant increase in TAPSE (t = 2.78 (Sforza et al. 2000), *p* = 0.03) for *ISI*1_High_ compared to SHAM, whereas there was no significant difference between *ISI*1_Low_ and SHAM (t = 1.47 (Sforza et al. 2000), *p* = 0.32). Therefore, *ISI1*_High_ significantly increased post‐sleep right‐ventricular systolic function compared to SHAM. Investigating whether the cardiovascular iAUC response can predict these changes in TAPSE, we found a significant positive linear repeated measures correlation between the induced cardiovascular response and TAPSE (see Table 1), indicating increased right‐ventricular systolic function with increased cardiovascular response.

**TABLE 1:** Within‐subject relationship between post‐sleep echocardiography parameters and cardiovascular responses after detected slow waves. To quantify cardiovascular responses to slow waves we used the integrated area under the curve for the echocardiographic parameters left‐ventricular ejection fraction (LVEF), and tricuspid annular plane systolic excursion (TAPSE), or peak‐to‐peak distance for the E/e’ ratio. The analysis involved 15 subjects for instantaneous heart rate (IHR) and 17 subjects over three nights for diastolic (DBP) and systolic blood pressure (SBP). Repeated measures linear correlations were computed to link transthoracic echocardiography parameters with the iAUC of the averaged cardiovascular responses from slow waves that occurred during a stimulation ON window of all conditions.

	IHR		SBP		DBP	
	*p*	*r*	*p*	*r*	*p*	*r*
LVEF	0.0037**	0.46	0.00047**	0.56	0.00099**	0.53
E/e’	0.011	−0.45	0.097	−0.29	0.042	−0.35
TAPSE	0.0043*	0.50	0.0069*	0.45	0.0028**	0.49

** Indicates significance after conservative Bonferroni correction (*p* < 0.0042).

* Indicates trend‐level significance after Bonferroni correction (*p* < 0.0084).

### 3.3 Type I and Type II Slow Waves Are Linked to Different Heart Rate and Blood Pressure Responses During Unstimulated Sleep

In the following step, our aim was to gain a deeper understanding of whether distinct slow waves are also occurring alongside distinct cardiovascular responses, independent of stimulation windows. Few studies proposed that strongly synchronized slow waves, such as K‐complexes, evoke a cardiovascular activation response (de Zambotti et al. 2016; Tank et al. 2003). Therefore, we classified the detected slow waves into type I and type II (see Methods) during periods of unstimulated sleep (SHAM condition, ON + OFF windows and slow waves occurring outside any stimulation window) and investigated the cardiovascular responses associated with each type of slow wave. The cardiovascular responses occurring within the 18 s following the onset of each slow wave were normalized by dividing by the values from the 2 s preceding each onset. Subsequently, we averaged these normalized cardiovascular responses for each type of slow wave within each subject, and then across all participants. As illustrated in Figure 4, the IHR exhibited a rapid, almost symmetrical increase and decrease in response. In contrast, type II slow waves elicited a relatively weaker response across all cardiovascular metrics. In addition, our finding indicated a more pronounced cardiovascular blood pressure response with type I slow waves, peaking approximately 5 s after the wave onset. There were statistically significant differences (*p* < 0.05) between type I and II slow waves in all cardiovascular metrics.

### 3.4 Auditory Stimulation Increases the Occurrence of Type I and Type II Slow Waves With Distinct Temporal Dynamics

Because a strengthened cardiovascular response during sleep predicts post‐sleep cardiac function (see Figure 3 and Table 1) we wanted to investigate whether auditory slow wave stimulation induces both types of slow waves similarly. Therefore, we identified occurrences of either type I or type II slow waves during the stimulation windows (ON and OFF) of all nights to calculate the likelihood of both types of slow waves occurring as a function of time during the stimulation window. The likelihood of each type was calculated by summing each of their occurrences, multiplying by 100, and then dividing these values against the total number of stimulation windows.

Figure 5 reveals that type I and type II slow wave likelihood differs in response to auditory stimulation. In the ON window, type II slow waves demonstrated an increase in wave likelihood in response to each tone played as stimulation was delivered in a rhythmic 1‐Hz manner. Notably, the initial tones induced on average a higher type II wave likelihood than the later tones, suggesting that the first tones are more effective than the last ones in evoking a slow wave. Of note, the first decreasing drop at the start of the stimulation windows in type II slow waves is associated with the up‐phase stimulation of ongoing slow waves (see Figure 5). For type I slow waves, we observed a pronounced increase in wave likelihood to auditory stimulation primarily at the onset of the stimulation window compared to the SHAM condition. Type I likelihood is not displaying a rhythmic response as observed for type II slow waves. Furthermore, the occurrence of type I slow waves is lower compared to type II slow waves.

## 4 Discussion

Our work uncovers that post‐sleep cardiac function is predicted by the strength of cardiovascular responses induced alongside slow waves. First, we confirmed our previous work (Huwiler et al. 2023; Huwiler et al. 2022) showing that auditory slow wave stimulation evokes a cardiovascular activation response, resembling cardiovascular pulsations. We then demonstrate that the strength of these cardiovascular pulsations is correlated with TTE‐derived cardiac left‐ventricular and right‐ventricular function the next morning. We further found that type I and type II slow waves are not only functionally distinct based on EEG‐based wave morphology, timing, and synchronization processes (Bernardi et al. 2018; Siclari et al. 2014), but also signify unique brain and body “micro‐states” during sleep that may be differently involved in promoting cardiac function. Specifically, both slow wave types are associated with distinct strengths of cardiovascular pulsations. Therefore, we provide mechanistic insights into how these two slow wave types are related to post‐sleep cardiac function. Lastly, we showed that auditory slow wave stimulation enhances the likelihood of both type I and II slow waves compared to the SHAM control condition, although the two types exhibit different underlying temporal dynamics. Overall, our findings suggest a potential pathway on how slow wave stimulation may promote cardiac function during sleep.

### 4.1 Type I Slow Waves Align With Strengthened Cortico‐Cardiovascular Coupling

To date, there is only limited evidence highlighting the importance of deep NREM sleep and slow waves in promoting cardiovascular health (Grimaldi et al. 2019; de Zambotti et al. 2016; de Zambotti et al. 2018; Sayk et al. 2010). We here describe a mechanistic explanation of how slow waves may increase cardiac function by demonstrating that the strength of cardiovascular pulsations induced alongside slow waves is correlated to enhancements in cardiac functions after sleep (see Figure 3 and Table 1). More specifically, the average cortico‐cardiovascular coupling (increased cardiovascular pulsations occurring alongside slow waves) during sleep slow waves predicts these cardiac improvements. Additionally, this cortico‐cardiovascular coupling is stronger in type I slow waves compared to type II slow waves (see Figure 4). However, because type II slow waves often occur in trains of slow waves, we cannot fully disentangle isolated effects of one slow wave on cardiovascular coupling. However, our data points towards potentially stronger implications of type I slow waves on post‐sleep cardiac functions than type II slow waves.

We furthermore show a cardiovascular activation as a response concurrent with type I slow waves, which is similar to the cardiovascular response previously reported with K‐complexes (de Zambotti et al. 2016; Sforza et al. 2000; Tank et al. 2003). Additionally, De Zambotti et al. (de Zambotti et al. 2016) demonstrated that played tones without eliciting a K‐complex did not evoke a biphasic cardiac response. They found that both naturally occurring and tone‐evoked K‐complexes elicit similar cardiac responses. Hence, an observable K‐complex in the sleep EEG serves as a necessary predictor or initiator for a cardiovascular response to manifest. Our study confirms the biphasic heart rate response observed in both stimulated and spontaneous type I slow waves comparable to K‐complexes (de Zambotti et al. 2016; de Zambotti et al. 2018). Therefore, our findings support the hypothesis of an involvement of K‐complexes or type I slow waves in blood pressure (Tank et al. 2003) and cardiac (de Zambotti et al. 2016) regulation, possibly directly influencing cardiovascular function during sleep (de Zambotti et al. 2016; Sforza et al. 2000; Tank et al. 2003; de Zambotti et al. 2018). Thus, we provide evidence that the mechanisms underlying the generation of type I slow waves are also modulating the cardiovascular system during sleep (de Zambotti et al. 2016), highlighting their importance as a fundamental sleep element associated with promoting cardiovascular health (Huwiler et al. 2023). Moreover, our findings propose a potential mechanistic explanation contributing to aging as the major risk factor for cardiovascular diseases (North and Sinclair 2012), because aging is associated with a strong decline in K‐complex density (Crowley et al. 2002). Altogether, auditory slow wave stimulation protocols aiming to increase cardiovascular function should focus on increasing type I slow wave occurrence and essentially its stronger associated cortico‐cardiovascular coupling.

### 4.2 Type I and Type II Slow Waves Signify Unique Brain and Body Microstates

We observed coupled cardiovascular pulsations being evoked in association with both types of slow waves. The increase in response in type I slow waves could be caused by the stronger activation of brain structures that are also involved in autonomic modulation of the cardiovascular system. On an EEG morphological level, the two distinct types of slow waves have been proposed to originate from distinct generation and synchronization mechanisms within the central nervous system (Bernardi et al. 2018). More specifically, type I slow waves have been suggested to manifest through subcortico‐cortical synchronization processes, whereas type II slow waves emerge through cortico‐cortical synchronization (Bernardi et al. 2018; Siclari et al. 2014). Our observed stronger cortico‐cardiovascular coupling in type I slow waves supports the idea of an enhanced subcortical involvement because the processes underlying type I slow waves likely activate the sympathoexcitatory locus coeruleus (Bellesi et al. 2014), a small nucleus in the brain stem serving as the noradrenergic center of the brain. Supporting this idea, we recently showed a pupil dilation occurring alongside K‐complexes (Carro‐Domínguez et al. 2025), pointing towards a potential increase in locus coeruleus activity concurrent with highly synchronized slow waves (Murphy et al. 2014). Additionally, K‐complexes coincide with spikes in muscle sympathetic nerve activity (Tank et al. 2003; Somers et al. 1993), also supporting the stronger cardiovascular activation response compared to type II slow waves, aligning with an observed autonomic activation response after scored K‐complexes (Tank et al. 2003; Johnson and Karpan 1968). This activation response could even be attributed to dampening the baroreflex, leading to sleep continuity (Tank et al. 2003). Nevertheless, this increased cardiovascular activation response during sleep could also reflect elevated sympathetic activity, which, if sustained, might be harmful to patients. Altogether, type I and type II slow waves are not only distinguishable by cortical characteristics, but also by their different cardiovascular activation responses.

### 4.3 Slow Wave Stimulation as a Potential Therapeutic Tool to Enhance Cardiac Function

Our findings emphasize the potential of slow wave stimulation to strengthen cortico‐cardiovascular coupling as a tool for increasing cardiovascular function. We observed a significant correlation between the cortico‐cardiovascular coupling and two markers estimating left‐ventricular systolic function. On the one hand, strain maps the complex contraction of the left‐ventricular myocardium (Sugimoto et al. 2017), and has been established as an important marker indicating deformation of the left ventricle and for assessing left‐ventricular systolic function (Bergler‐Klein 2015; McDonagh et al. 2021). Left‐ventricular global longitudinal strain was found to be independent of HR (Fredholm et al. 2017), and therefore, as blood pressure between conditions did not differ during a measurement closest to the TTE measurement, this increase in strain can be explained by our sleep intervention. On the other hand, LVEF, the current standard clinical method to assess left‐ventricular systolic function, represents an important parameter for the primary and secondary prevention of cardiac diseases (Wang et al. 2003; Breathett et al. 2016). In addition to systolic function, we also found a significant correlation between the cortico‐cardiovascular coupling and decreased E/e’ ratio. The left‐ventricular diastolic function reflects relaxation and stiffness of the myocardium (Nagueh et al. 2016; Owan et al. 2006). Particularly, an E/e’ ratio > 14 has been related to abnormal diastolic function of the heart (Nagueh et al. 2016). In addition to left‐ventricular function, we also found a positive correlation between the cardiovascular response during sleep and TAPSE. TAPSE has been associated with increased risks for right‐ventricular myocardial infarction (Rallidis et al. 2014), and is proposed to be an important predictor for survival in cardiopulmonary diseases (Haddad et al. 2008). Given these strong within‐subject correlations between the strength of the cortico‐cardiovascular coupling during sleep and increases in cardiac function the next morning, promoting these cardiovascular pulsations could represent an important preventative pathway in maintaining cardiovascular health.

Given that this cortico‐cardiovascular coupling is enhanced in type I slow waves compared to type II slow waves, future stimulation protocols could focus on enhancing type I slow waves such as K‐complexes. However, most K‐complexes occur spontaneously during NREM sleep, without any external cause (Monstad and Guilleminault 1999; De Gennaro et al. 2000; Halász 2005), but K‐complexes can also be evoked through somatosensory stimulation such as auditory stimulation (Bellesi et al. 2014). We show here that both, type I and type II slow waves are enhanced through auditory stimulation (see Figure 5). Type I slow waves were evoked only at the start of each stimulation window, potentially because of a refractory period that has been reported for K‐complexes (Bernardi et al. 2018; Bastien and Campbell 1994; Halász 2016). Type II slow waves, on the other hand, can be evoked or even entrained with each single presented tone. However, both types of slow waves likely contribute to cardiac fluctuations and therefore, may even increase heart rate variability (de Zambotti et al. 2016), but to a different extent. Altogether, our findings underscore the potential of sleep slow wave stimulation as a therapeutic tool to maintain and even increase cardiovascular function. By differentiating the responses of type I and II slow waves, our study contributes to a deeper understanding of how slow waves may be strategically influenced to optimize cardiovascular health by strengthening cortico‐cardiovascular coupling. Therefore, in order to improve cardiovascular health, we propose to develop (auditory) sleep stimulation protocols to optimize the occurrence of these cardiovascular fluctuations and increase the cortical‐cardiovascular coupling, by optimizing type I slow wave occurrence.

### 4.4 Cortico‐Cardiovascular Coupling May Deteriorate in Neurodegenerative Disorders

Besides cardiovascular diseases, Alzheimer's disease represents another aging disease with shared risk factors that are associated with changes in sleep (De Gennaro et al. 2017; de Bruijn and Ikram 2014; Himali et al. 2023). Recent studies underscore the crucial role of sleep, particularly NREM sleep, in facilitating the removal of metabolic waste from the brain through the glymphatic system (Fultz et al. 2019; Vijayakrishnan Nair et al. 2023). Mainly with a focus on improving memory functions, auditory stimulation during sleep has been employed to increase slow waves (Van den Bulcke et al. 2023; Wunderlin et al. 2023), and has been related to a more favorable amyloid plasma response. Recently it has been shown that plasma proteomic signatures indicating accelerated heart aging predict Alzheimer's disease progression (Oh et al. 2023), again emphasizing a tight link between sleep, cardiovascular function, and neurodegenerative disorders such as Alzheimer's disease. Interestingly, the process of metabolic brain clearance seems to be driven by vasomotor pulsations throughout the brain (Helakari et al. 2022), which are potentially directed by autonomic arousals (Picchioni et al. 2022). These pulsations, in turn, amplify the flow of cerebrospinal fluid (Bojarskaite et al. 2023). The here‐described and observed cortico‐cardiovascular coupling is consistent with the previously reported autonomic arousals (Picchioni et al. 2022) to cleanse the brain. Therefore, the decrease in strength of this coupling could represent an important mechanism of how the degenerating brain in Alzheimer's disease pre‐clinical stage results in the accumulation of amyloid plaque. Essentially, increasing this cortico‐cardiovascular coupling during sleep by evoking type I slow waves or K‐complexes might increase cerebrospinal fluid flow and thereby decelerate amyloid accumulation. Future studies applying auditory sleep stimulation to explore the functional connection of slow waves and amyloid‐beta should also assess the strength of the cardiovascular pulsations alongside slow waves to investigate whether these autonomic activation responses are truly a driver for cerebrospinal fluid flow and amyloid accumulation.

### 4.5 Limitations

Our study only investigated healthy male participants between 30 and 60 years of age. The female menstrual cycle has been shown to influence sleep (Baker and Driver 2007) and also HR during sleep (Alzueta et al. 2022), and menopause generally occurs in women within our targeted age range, coinciding with sleep modifications and disturbances (Gómez‐Santos et al. 2016). These hormonal differences would make it difficult to investigate the effects of slow waves on cardiovascular functions across sessions and to generalize findings across participants. Therefore, for this initial study, we decided to only include male participants. Nevertheless, future large‐scale studies should also explore type I and type II slow waves and their relationship with cardiovascular function across sex. Moreover, the number of participants in this study appears relatively low. While this number is comparable to other interventional sleep studies (Ngo et al. 2013; Ngo et al. 2015; Besedovsky et al. 2017), a larger scale study applying auditory stimulation across multiple nights and in a broader and more heterogeneous population sample is needed to characterize long‐term effects. It is especially important to rule out the possibility that such a longer‐term increase in the cortico‐cardiovascular coupling influences sympathetic tone, as this would be similar to the described pathomechanism relating transportation noise pollution to increased cardiovascular risks (Münzel et al. 2024). In this study, we tested healthy male participants. However, to verify the prospective clinical application for enhancing cardiac function, in individuals with heightened cardiovascular risks or existing cardiovascular conditions, it is essential to first establish the preventive and therapeutic effects associated with increased cortico‐cardiovascular coupling during sleep. Lastly, the automatic EEG data preprocessing and rejection pipeline which we used has not been validated on sleep EEG data that may potentially lead to rejecting unwanted components of the EEG data. However, this processing was mainly used as a guide with manual confirmation, and the number of rejected components is consistent with previous work (Bernardi et al. 2018; Siclari et al. 2014) which this pipeline was based on.

## 5 Conclusion

In this study, we present insights into the cardiovascular implications of slow waves during sleep. Our findings reveal that auditory slow wave stimulation strengthens cortico‐cardiovascular coupling, and its strength predicts cardiac function. Particularly type I slow waves, including K‐complexes, co‐occur with stronger cardiovascular pulsations compared to type II slow waves, indicating that both types of slow waves represent unique brain and body micro‐states. Moreover, we showed that auditory stimulation differently intensifies both types of slow waves. By establishing the connection between slow wave types and their cardiovascular responses, our study marks a significant advancement in decoding the complex relationship between sleep physiology and cardiovascular function.

## Author Contributions

Conceptualization: Giulia Alessandrelli, Stephanie Huwiler, Reto Huber, Nicole Wenderoth, Christian Schmied, and Caroline Lustenberger. Methodology: Giulia Alessandrelli, Giulio Bernardi, Stephanie Huwiler, and Caroline Lustenberger. Software: Giulia Alessandrelli, Francesca Siclari, Giulio Bernardi, and Stephanie Huwiler. Validation: Giulia Alessandrelli. Formal analysis: Giulia Alessandrelli, and Stephanie Huwiler. Investigation: Stephanie Huwiler, Manuel Carro‐Domínguez, Francesca Siclari, Rossella Sala, Florent Aziri, Anna Trippel, Susanne Markendorf, David Niederseer, Philipp Bohm, and Christian Schmied. Resources: Nicole Wenderoth, Christian Schmied, and Caroline Lustenberger. Data curation: Giulia Alessandrelli and Stephanie Huwiler. Visualization: Giulia Alessandrelli and Stephanie Huwiler. Supervision: Nicole Wenderoth and Caroline Lustenberger. Writing – original draft: Giulia Alessandrelli, Stephanie Huwiler, and Caroline Lustenberger. Writing – review and editing: Giulia Alessandrelli, Stephanie Huwiler, Giulio Bernardi, Manuel Carro‐Domínguez, Francesca Siclari, Rossella Sala, Florent Aziri, Anna Trippel, Susanne Markendorf, David Niederseer, Philipp Bohm, Pietro Cerveri, Francesca Siclari, Reto Huber, Nicole Wenderoth, Christian Schmied, and Caroline Lustenberger. Project administration: Stephanie Huwiler and Caroline Lustenberger. Funding acquisition: Nicole Wenderoth, Pietro Cerveri, and Caroline Lustenberger.

## Funding

This work was supported through the Swiss National Science Foundation (PZ00P3_179795 to C.L., 32003B_207719 to N.W., and 320030_179443 to R.H.), the European Research Council (ERC; Starting Grant 948891 to G.B.), the Italian Ministry of University and Research—PON Program Green and Innovation (2014–2020, to P.C.), and the National Research Foundation, Prime Minister's Office, Singapore under its Campus for Research Excellence and Technological Enterprise (CREATE) program (FHT). This work was conducted as part of the SleepLoop Flagship of Hochschulmedizin Zürich.

## Ethics Statement

The study was approved by the Cantonal Ethics Committee Zurich.

## Consent

All participants provided written informed consent before participation and received monetary compensation for their participation.

## Conflicts of Interest

Reto Huber is a founder and shareholder of tosoo AG. The other authors declare no conflicts of interest.

## Supporting information


**Figure S1:** CONSORT Flow Diagram for trial overview.
**Figure S2:** Visual representation of type I and II slow waves. Figures A and B provide a visual representation of slow wave types during an unstimulated night. The black signal shows the single‐channel EEG derived from a multi‐channel recording (in grey) after undergoing the pre‐processing steps detailed in the methodology. The vertical dotted lines indicate the start and end of each slow wave. (A) illustrates a type I slow wave, while (B) shows a type II slow wave, both recorded from a 30‐year‐old male subject.
**Figure S3:** Effects of auditory stimulation on systolic blood pressure (BP) in the morning. Data is presented as mean ± standard error of the mean for *n* = 18 participants. *p*‐values have been computed based on a linear‐mixed effect model with the fixed factor Condition (*ISI1*_High_, *ISI1*_Low_, or SHAM) and random factor subject.
**Figure S4:** Effects of auditory stimulation on echocardiography parameter estimating right‐ventricular systolic function. Right‐ventricular tricuspid annular plane systolic excursion (TAPSE) is presented as mean ± standard error of the mean for *n* = 18 participants. *p*‐values have been computed based on a linear‐mixed effect model with the fixed factor Condition (*ISI1*_High_, *ISI1*_Low_, or SHAM) and random factor subject. Post hoc comparison between conditions has been computed through paired t‐tests and corrected for multiple comparisons by applying the Bonferroni correction. *: *p* < 0.05.
**Figure S5:** Panels A–B show the waveforms of representative slow waves for Type I (A) and Type II (B), with the bold line indicating the average across electrodes. Panels C–E illustrate the corresponding scalp topographies: (C) Type I, (D) Type II, and (E) the relative difference. Both subtypes exhibit a frontal maximum, with Type I displaying stronger and more widespread activity, consistent with previous reports.
**Figure S6:** Cardiovascular responses to type I and type II slow waves during the ISI_Low_ condition. Event‐related changes in instantaneous heart rate (IHR), systolic blood pressure (SBP), and diastolic blood pressure (DBP) are shown time‐locked to the occurrence of Type I (blue) and Type II (orange) slow waves. Shaded areas represent the standard error of the mean (SEM). As in the SHAM condition (Figure 4, main text), Type I slow waves are associated with larger transient cardiovascular responses than Type II waves. Statistical comparisons were conducted using the same within‐subject tests as in the main figure (paired‐samples t‐tests across participants for each metric).
**Figure S7:** Cardiovascular responses to Type I and Type II slow waves during the ISI_High_ condition. Event‐related changes in IHR, SBP, and DBP time‐locked to Type I (blue) and Type II (orange) slow waves. The overall pattern replicates the SHAM and ISI_Low_ conditions, with Type I slow waves eliciting stronger cardiovascular responses than Type II. Shaded areas denote SEM. Statistical analysis followed the same procedure as in the main figure, confirming the robustness of the observed differences.
**Table S1:** Included number of slow waves (mean + ‐SEM).
**Table S2:** Included number of Type I and Type II slow waves (mean + ‐SEM).

## Data Availability

Source data required to reproduce the main figures and main conclusions of the manuscript will be made available in the future.

## References

[jsr70354-bib-0001] Alzueta, E. , M. De Zambotti , H. Javitz , et al. 2022. “Symptoms Across the Menstrual Cycle With the Oura Ring in Healthy Women Tracking Sleep, Temperature, Heart Rate, and Daily Symptoms Across the Menstrual Cycle With the Oura Ring in Healthy Women.” International Journal of Women's Health 14: 491–503. 10.2147/IJWH.S341917. PMC9005074 35422659

[jsr70354-bib-0002] Baker, F. C. , and H. S. Driver . 2007. “Circadian Rhythms, Sleep, and the Menstrual Cycle.” Sleep Medicine 8, no. 6: 613–622. 10.1016/j.sleep.2006.09.011. 17383933

[jsr70354-bib-0003] Bastien, C. , and K. Campbell . 1994. “Effects of Rate of Tone‐Pip Stimulation on the Evoked K‐Complex.” Journal of Sleep Research 3, no. 2: 65–72. 10.1111/j.1365-2869.1994.tb00109.x. 10607110

[jsr70354-bib-0004] Bates, D. , M. Mächler , B. Bolker , and S. Walker . 2015. “Fitting Linear Mixed‐Effects Models Using lme4.” Journal of Statistical Software 67, no. 1: 1–48. 10.18637/jss.v067.i01.

[jsr70354-bib-0005] Behar, J. A. , A. A. Rosenberg , I. Weiser‐Bitoun , et al. 2018. “PhysioZoo: A Novel Open Access Platform for Heart Rate Variability Analysis of Mammalian Electrocardiographic Data.” Frontiers in Physiology 9: 1–14. 10.3389/fphys.2018.01390. PMC6180147 30337883

[jsr70354-bib-0006] Bellesi, M. , B. A. Riedner , G. N. Garcia‐Molina , C. Cirelli , and G. Tononi . 2014. “Enhancement of Sleep Slow Waves: Underlying Mechanisms and Practical Consequences.” Frontiers in Systems Neuroscience 8, no. 2: 82–88. 10.3389/fnsys.2014.00208. PMC4211398 25389394

[jsr70354-bib-0007] Bergler‐Klein, J. 2015. “Strain and Left Ventricular Volumes for Predicting Cardiotoxicity: A Life‐Saving Approach in Anthracycline cancer Treatment?” European Heart Journal Cardiovascular Imaging 16, no. 9: 968–969. 10.1093/ehjci/jev168. 26206463

[jsr70354-bib-0008] Bernardi, G. , and F. Siclari . 2019. “Local Patterns of Sleep and Wakefulness.” In In: Handbook of Sleep Research, 33–47. Elsevier. 10.1016/b978-0-12-813743-7.00003-7.

[jsr70354-bib-0009] Bernardi, G. , F. Siclari , G. Handjaras , B. A. Riedner , and G. Tononi . 2018. “Local and Widespread Slow Waves in Stable NREM Sleep: Evidence for Distinct Regulation Mechanisms.” Frontiers in Human Neuroscience 12: 1–13. 10.3389/fnhum.2018.00248. PMC6018150 29970995

[jsr70354-bib-0010] Besedovsky, L. , H. V. V. Ngo , S. Dimitrov , C. Gassenmaier , R. Lehmann , and J. Born . 2017. “Auditory Closed‐Loop Stimulation of EEG Slow Oscillations Strengthens Sleep and Signs of Its Immune‐Supportive Function.” Nature Communications 8, no. 1: 1984. 10.1038/s41467-017-02170-3. PMC5719447 29215045

[jsr70354-bib-0011] Bigdely‐Shamlo, N. , T. Mullen , C. Kothe , K. M. Su , and K. A. Robbins . 2015. “The PREP Pipeline: Standardized Preprocessing for Large‐Scale EEG Analysis.” Frontiers in Neuroinformatics 9: 16. 10.3389/fninf.2015.00016. PMC4471356 26150785

[jsr70354-bib-0012] Bojarskaite, L. , A. Vallet , D. M. Bjørnstad , et al. 2023. “Sleep Cycle‐Dependent Vascular Dynamics in Male Mice and the Predicted Effects on Perivascular Cerebrospinal Fluid Flow and Solute Transport.” Nature Communications 14, no. 1: 953. 10.1038/s41467-023-36643-5. PMC9941497 36806170

[jsr70354-bib-0013] Breathett, K. , L. A. Allen , J. Udelson , G. Davis , and M. Bristow . 2016. “Changes in Left Ventricular Ejection Fraction Predict Survival and Hospitalization in Heart Failure With Reduced Ejection Fraction.” Circulation. Heart Failure 9, no. 10. 10.1161/CIRCHEARTFAILURE.115.002962. PMC5082710 27656000

[jsr70354-bib-0015] Cappuccio, F. P. , D. Cooper , L. D'Elia , P. Strazzullo , and M. A. Miller . 2011. “Sleep Duration Predicts Cardiovascular Outcomes: A Systematic Review and meta‐Analysis of Prospective Studies.” European Heart Journal 32, no. 12: 1484–1492. 10.1093/eurheartj/ehr007. 21300732

[jsr70354-bib-0016] Carro‐Domínguez, M. , S. Huwiler , S. Oberlin , et al. 2025. “Pupil Size Reveals Arousal Level Fluctuations in Human Sleep.” Nature Communications 16, no. 1: 2070. 10.1038/s41467-025-57289-5. PMC11871316 40021662

[jsr70354-bib-0017] Crowley, K. , J. Trinder , Y. Kim , M. Carrington , and I. M. Colrain . 2002. “The Effects of Normal Aging on Sleep Spindle and K‐Complex Production.” Clinical Neurophysiology 113, no. 10: 1615–1622. 10.1016/S1388-2457(02)00237-7. 12350438

[jsr70354-bib-0014] de Bruijn, R. F. A. G. , and M. A. Ikram . 2014. “Cardiovascular Risk Factors and Future Risk of Alzheimer's Disease.” BMC Medicine 12, no. 1: 1–9. 10.1186/s12916-014-0130-5. PMC4226863 25385322

[jsr70354-bib-0018] De Gennaro, L. , M. Ferrara , and M. Bertini . 2000. “The Spontaneous K‐Complex During Stage 2 Sleep: Is It the Forerunner of delta Waves?” Neuroscience Letters 291, no. 1: 41–43. 10.1016/S0304-3940(00)01366-5. 10962149

[jsr70354-bib-0019] De Gennaro, L. , M. Gorgoni , F. Reda , et al. 2017. “The Fall of Sleep K‐Complex in Alzheimer Disease.” Scientific Reports 7, no. 1: 39688. 10.1038/srep39688. PMC5206737 28045040

[jsr70354-bib-0020] Delorme, A. , and S. Makeig . 2004. “EEGLAB: An Open Source Toolbox for Analysis of Single‐Trial EEG Dynamics Including Independent Component Analysis.” Journal of Neuroscience Methods 134, no. 1: 9–21. 10.1016/j.jneumeth.2003.10.009. 15102499

[jsr70354-bib-0021] Delorme, A. , T. Sejnowski , and S. Makeig . 2007. “Enhanced Detection of Artifacts in EEG Data Using Higher‐Order Statistics and Independent Component Analysis.” NeuroImage 34: 1443–1449. 10.1016/j.neuroimage.2006.11.004. PMC2895624 17188898

[jsr70354-bib-0022] Diep, C. , S. Ftouni , S. P. A. Drummond , G. Garcia‐Molina , and C. Anderson . 2022. “Heart Rate Variability Increases Following Automated Acoustic Slow Wave Sleep Enhancement.” Journal of Sleep Research 31: 1–6. 10.1111/jsr.13545. 35080060

[jsr70354-bib-0023] Ferster, M. L. , C. Lustenberger , and W. Karlen . 2019. “Configurable Mobile System for Autonomous High‐Quality Sleep Monitoring and Closed‐Loop Acoustic Stimulation.” IEEE Sensors Letters 3, no. 5: 1–1. 10.1109/LSENS.2019.2914425.

[jsr70354-bib-0024] Fredholm, M. , K. Jörgensen , E. Houltz , and S. E. Ricksten . 2017. “Load‐Dependence of Myocardial Deformation Variables – A Clinical Strain‐Echocardiographic Study.” Acta Anaesthesiologica Scandinavica 61, no. 9: 1155–1165. 10.1111/aas.12954. 28804896

[jsr70354-bib-0025] Fultz, N. E. , G. Bonmassar , K. Setsompop , et al. 2019. “Coupled Electrophysiological, Hemodynamic, and Cerebrospinal Fluid Oscillations in Human Sleep.” Science 366, no. 6465: 628–631. 10.1126/science.aax5440. PMC7309589 31672896

[jsr70354-bib-0026] Gómez‐Santos, C. , C. B. Saura , J. A. R. Lucas , P. Castell , J. A. Madrid , and M. Garaulet . 2016. “Menopause Status Is Associated With Circadian‐ and Sleep‐Related Alterations.” Menopause 23, no. 6: 682–690. 10.1097/GME.0000000000000612. 27093617

[jsr70354-bib-0027] Grandner, M. A. , L. Hale , M. Moore , and N. P. Patel . 2010. “Mortality Associated With Short Sleep Duration: The Evidence, the Possible Mechanisms, and the Future.” Sleep Medicine Reviews 14, no. 3: 191–203. 10.1016/j.smrv.2009.07.006. PMC2856739 19932976

[jsr70354-bib-0028] Greenlund, I. M. , C. A. Smoot , and J. R. Carter . 2021. “Sex Differences in Blood Pressure Responsiveness to Spontaneous K‐Complexes During Stage II Sleep.” Journal of Applied Physiology 130: 491–497. 10.1152/japplphysiol.00825.2020. PMC7948112 33300855

[jsr70354-bib-0029] Grimaldi, D. , N. A. Papalambros , K. J. Reid , et al. 2019. “Strengthening Sleep‐Autonomic Interaction via Acoustic Enhancement of Slow Oscillations.” Sleep 42: 1–11. 10.1093/sleep/zsz036. PMC7729207 30753650

[jsr70354-bib-0030] Haddad, F. , R. Doyle , D. J. Murphy , and S. A. Hunt . 2008. “Right Ventricular Function in Cardiovascular Disease, Part II: Pathophysiology, Clinical Importance, and Management of Right Ventricular Failure.” Circulation 117, no. 13: 1717–1731. 10.1161/CIRCULATIONAHA.107.653584. 18378625

[jsr70354-bib-0031] Halász, P. 2005. “K‐Complex, a Reactive EEG Graphoelement of NREM Sleep: An Old Chap in a New Garment.” Sleep Medicine Reviews 9, no. 5: 391–412. 10.1016/j.smrv.2005.04.003. 16122950

[jsr70354-bib-0032] Halász, P. 2016. “The K‐Complex as a Special Reactive Sleep Slow Wave ‐ A Theoretical Update.” Sleep Medicine Reviews 29: 34–40. 10.1016/j.smrv.2015.09.004. 26606317

[jsr70354-bib-0033] Helakari, H. , V. Korhonen , S. C. Holst , et al. 2022. “Human NREM Sleep Promotes Brain‐Wide Vasomotor and Respiratory Pulsations.” Journal of Neuroscience 42, no. 12: 2503–2515. 10.1523/JNEUROSCI.0934-21.2022. PMC8944230 35135852

[jsr70354-bib-0034] Himali, J. J. , A. A. Baril , M. G. Cavuoto , et al. 2023. “Association Between Slow‐Wave Sleep Loss and Incident Dementia.” JAMA Neurology 80, no. 12: 1326–1333. 10.1001/jamaneurol.2023.3889. PMC10616771 37902739

[jsr70354-bib-0035] Huwiler, S. , M. Domínguez , S. Huwyler , et al. 2022. “Effects of Auditory Sleep Modulation Approaches on Brain Oscillatory and Cardiovascular Dynamics.” Sleep 45, no. 9: 1–36. 10.1093/sleep/zsac155. PMC9453626 35793672

[jsr70354-bib-0036] Huwiler, S. , M. Carro‐Domínguez , F. M. Stich , et al. 2023. “Auditory Stimulation of Sleep Slow Waves Enhances Left Ventricular Function in Humans.” European Heart Journal 44, no. 40: 4288–4291. 10.1093/eurheartj/ehad630. PMC10590124 37794725

[jsr70354-bib-0037] Johnson, L. , and W. E. Karpan . 1968. “Autonomic Correlates of the Spontaneous K‐Complex.” Psychophysiology 4, no. 4: 444–452. 10.1111/j.1469-8986.1968.tb02785.x. 5662814

[jsr70354-bib-0038] Klaeboe, L. G. , and T. Edvardsen . 2019. “Echocardiographic Assessment of Left Ventricular Systolic Function.” Journal of Echocardiography 17, no. 1: 10–16. 10.1007/s12574-018-0405-5. 30390189

[jsr70354-bib-0039] Kohansieh, M. , and A. N. Makaryus . 2015. “Sleep Deficiency and Deprivation Leading to Cardiovascular Disease.” International Journal of Hypertension 2015: 1–5. 10.1155/2015/615681. PMC4606167 26495139

[jsr70354-bib-0040] Lustenberger, C. , M. L. Ferster , S. Huwiler , et al. 2022. “Auditory Deep Sleep Stimulation in Older Adults at Home: A Randomized Crossover Trial.” Communication & Medicine 2, no. 1: 30. 10.1038/s43856-022-00096-6. PMC9053232 35603302

[jsr70354-bib-0041] Makeig, S. , A. J. Bell , T. P. Jung , and T. J. Sejnowski . 1995. Independent Component Analysis of Electroencephalographic Data. In: Proceedings of the 8th International Conference on Neural Information Processing Systems. NIPS’95, 145–151. MIT Press.

[jsr70354-bib-0042] Makeig, S. , S. Debener , J. Onton , and A. Delorme . 2004. “Mining Event‐Related Brain Dynamics.” Trends in Cognitive Sciences 8: 204–210. 10.1016/j.tics.2004.03.008. 15120678

[jsr70354-bib-0043] McDonagh, T. A. , M. Metra , M. Adamo , et al. 2021. “2021 ESC Guidelines for the Diagnosis and Treatment of Acute and Chronic Heart Failure.” European Heart Journal 42, no. 36: 3599–3726. 10.1093/eurheartj/ehab368. 34447992

[jsr70354-bib-0044] Monstad, P. , and C. Guilleminault . 1999. “Cardiovascular Changes Associated With Spontaneous and Evoked K‐ Complexes.” Neuroscience Letters 263: 211–213. 10.1016/S0304-3940(99)00142-1. 10213173

[jsr70354-bib-0045] Münzel, T. , M. Molitor , M. Kuntic , et al. 2024. “Transportation Noise Pollution and Cardiovascular Health.” Circulation Research 134, no. 9: 1113–1135. 10.1161/CIRCRESAHA.123.323584. 38662856

[jsr70354-bib-0046] Murphy, P. R. , R. G. O'Connell , M. O'Sullivan , I. H. Robertson , and J. H. Balsters . 2014. “Pupil Diameter Covaries With BOLD Activity in Human Locus Coeruleus.” Human Brain Mapping 35, no. 8: 4140–4154. 10.1002/hbm.22466. PMC6869043 24510607

[jsr70354-bib-0047] Nagueh, S. F. , O. A. Smiseth , C. P. Appleton , et al. 2016. “Recommendations for the Evaluation of Left Ventricular Diastolic Function by Echocardiography: An Update From the American Society of Echocardiography and the European Association of Cardiovascular Imaging.” Journal of the American Society of Echocardiography 29, no. 4: 277–314. 10.1016/j.echo.2016.01.011. 27037982

[jsr70354-bib-0048] Ngo, H. V. V. , T. Martinetz , J. Born , M. Mölle , and M. Molle . 2013. “Auditory Closed‐Loop Stimulation of the Sleep Slow Oscillation Enhances Memory.” Neuron 78, no. 3: 545–553. 10.1016/j.neuron.2013.03.006. 23583623

[jsr70354-bib-0049] Ngo, H. V. V. , A. Miedema , I. Faude , T. Martinetz , M. Mölle , and J. Born . 2015. “Driving Sleep Slow Oscillations by Auditory Closed‐Loop Stimulation—A Self‐Limiting Process.” Journal of Neuroscience 35, no. 17: 6630–6638. 10.1523/JNEUROSCI.3133-14.2015. PMC4412888 25926443

[jsr70354-bib-0050] North, B. J. , and D. A. Sinclair . 2012. “The Intersection Between Aging and Cardiovascular Disease.” Circulation Research 110, no. 8: 1097–1108. 10.1161/CIRCRESAHA.111.246876. PMC3366686 22499900

[jsr70354-bib-0051] Oh, H. S. H. , J. Rutledge , D. Nachun , et al. 2023. “Organ Aging Signatures in the Plasma Proteome Track Health and Disease.” Nature 624, no. 7990: 164–172. 10.1038/s41586-023-06802-1. PMC10700136 38057571

[jsr70354-bib-0052] Owan, T. E. , D. O. Hodge , R. M. Herges , S. J. Jacobsen , V. L. Roger , and M. M. Redfield . 2006. “Trends in Prevalence and Outcome of Heart Failure With Preserved Ejection Fraction.” New England Journal of Medicine 355, no. 3: 251–259. 10.1056/nejmoa052256. 16855265

[jsr70354-bib-0053] Picchioni, D. , P. S. Özbay , H. Mandelkow , et al. 2022. “Autonomic Arousals Contribute to Brain Fluid Pulsations During Sleep.” NeuroImage 249: 118888. 10.1016/j.neuroimage.2022.118888. PMC11395500 35017126

[jsr70354-bib-0054] Pion‐Tonachini, L. , K. Kreutz‐Delgado , and S. Makeig . 2019. “{ICLabel}: An Automated Electroencephalographic Independent Component Classifier, Dataset, and Website.” NeuroImage 198: 181–197. 10.1016/j.neuroimage.2019.05.026. PMC6592775 31103785

[jsr70354-bib-0055] R Core Team . 2013. “R: A Language and Environment for Statistical Computing.”

[jsr70354-bib-0056] Rallidis, L. S. , G. Makavos , and P. Nihoyannopoulos . 2014. “Right Ventricular Involvement in Coronary Artery Disease: Role of Echocardiography for Diagnosis and Prognosis.” Journal of the American Society of Echocardiography 27, no. 3: 223–229. 10.1016/j.echo.2013.12.001. 24412341

[jsr70354-bib-0057] Renard, Y. , F. Lotte , G. Gibert , et al. 2010. “OpenViBE: An Open‐Source Software Platform to Design, Test, and Use Brain‐Computer Interfaces in Real and Virtual Environments.” Presence (Cambridge, Mass) 19, no. 1: 35–53. 10.1162/pres.19.1.35.

[jsr70354-bib-0058] Rogasch, N. C. , C. Sullivan , R. H. Thomson , et al. 2017. “Analysing Concurrent Transcranial Magnetic Stimulation and Electroencephalographic Data: A Review and Introduction to the Open‐Source TESA Software.” NeuroImage 147: 934–951. 10.1016/j.neuroimage.2016.10.031. 27771347

[jsr70354-bib-0059] Sayk, F. , C. Teckentrup , C. Becker , et al. 2010. “Effects of Selective Slow‐Wave Sleep Deprivation on Nocturnal Blood Pressure Dipping and Daytime Blood Pressure Regulation.” American Journal of Physiology. Regulatory, Integrative and Comparative Physiology 298, no. 1: 191–197. 10.1152/ajpregu.00368.2009. 19907004

[jsr70354-bib-0060] Sforza, E. , C. Jouny , and V. Ibanez . 2000. “Cardiac Activation During Arousal in Humans: Further Evidence for Hierarchy in the Arousal Response.” Clinical Neurophysiology 111, no. 9: 1611–1619. 10.1016/S1388-2457(00)00363-1. 10964073

[jsr70354-bib-0061] Siclari, F. , J. J. LaRocque , B. R. Postle , et al. 2014. “Two Distinct Synchronization Processes in the Transition to Sleep: A High‐Density Electroencephalographic Study.” Sleep 37, no. 10: 1621–1637F. 10.5665/sleep.4070. PMC4173919 25197810

[jsr70354-bib-0062] Somers, V. K. , M. E. Dyken , A. L. Mark , and F. M. Abboud . 1993. “Sympathetic‐Nerve Activity During Sleep in Normal Subjects.” New England Journal of Medicine 328, no. 5: 303–307. 10.1056/NEJM199302043280502. 8419815

[jsr70354-bib-0063] Steriade, M. , I. Timofeev , and F. Grenier . 2001. “Natural Waking and Sleep States: A View From Inside Neocortical Neurons.” Journal of Neurophysiology 85, no. 5: 1969–1985. 10.1152/jn.2001.85.5.1969. 11353014

[jsr70354-bib-0064] Sugimoto, T. , R. Dulgheru , A. Bernard , et al. 2017. “Echocardiographic Reference Ranges for Normal Left Ventricular 2D Strain: Results From the EACVI NORRE Study.” European Heart Journal Cardiovascular Imaging 18, no. 8: 833–840. 10.1093/ehjci/jex140. 28637227

[jsr70354-bib-0065] Tank, J. , A. Diedrich , N. Hale , et al. 2003. “Relationship Between Blood Pressure, Sleep K‐Complexes, and Muscle Sympathetic Nerve Activity in Humans.” American Journal of Physiology. Regulatory, Integrative and Comparative Physiology 285: 208–214. 10.1152/ajpregu.00013.2003. 12793998

[jsr70354-bib-0066] Valenzuela, P. , A. Santos‐Lozano , A. Torres‐Barrán , et al. 2021. “Poor Self‐Reported Sleep Is Associated With Risk Factors for Cardiovascular Disease: A Cross‐Sectional Analysis in Half a Million Adults.” European Journal of Clinical Investigation 52: e13738. 10.1111/eci.13738. 34958676

[jsr70354-bib-0067] Vallat, R. 2018. “Pingouin: Statistics in Python.” Journal of Open Source Software 3, no. 31: 1026. 10.21105/joss.01026.

[jsr70354-bib-0068] Van den Bulcke, L. , A. M. Peeters , E. Heremans , et al. 2023. “Acoustic Stimulation as a Promising Technique to Enhance Slow‐Wave Sleep in Alzheimer's Disease: Results of a Pilot Study.” Journal of Clinical Sleep Medicine 19, no. 12: 2107–2112. 10.5664/JCSM.10778. PMC10692948 37593850

[jsr70354-bib-0069] Vijayakrishnan Nair, V. , B. R. Kish , P. L. Chong , et al. 2023. “Neurofluid Coupling During Sleep and Wake States.” Sleep Medicine 110: 44–53. 10.1016/j.sleep.2023.07.021. PMC11022242 37536211

[jsr70354-bib-0070] Wang, T. J. , J. C. Evans , E. J. Benjamin , D. Levy , E. C. LeRoy , and R. S. Vasan . 2003. “Natural History of Asymptomatic Left Ventricular Systolic Dysfunction in the Community.” Circulation 108, no. 8: 977–982. 10.1161/01.CIR.0000085166.44904.79. 12912813

[jsr70354-bib-0071] Wunderlin, M. , C. J. Zeller , S. R. Senti , et al. 2023. “Acoustic Stimulation During Sleep Predicts Long‐Lasting Increases in Memory Performance and Beneficial Amyloid Response in Older Adults.” Age and Ageing 52: 1–11. 10.1093/ageing/afad228. PMC10758173 38163288

[jsr70354-bib-0072] de Zambotti, M. , A. R. Willoughby , P. L. Franzen , D. B. Clark , F. C. Baker , and I. M. Colrain . 2016. “K‐Complexes: Interaction Between the Central and Autonomic Nervous Systems During Sleep.” Sleep 39, no. 5: 1129–1137. 10.5665/sleep.5770. PMC4835312 26856907

[jsr70354-bib-0073] de Zambotti, M. , J. Trinder , A. Silvani , I. M. Colrain , and F. C. Baker . 2018. “Dynamic Coupling Between the Central and Autonomic Nervous Systems During Sleep: A Review.” Neuroscience and Biobehavioral Reviews 90: 84–103. 10.1016/j.neubiorev.2018.03.027. PMC5993613 29608990

