# Supplementary material for: Cardiovascular Responses to Natural and Auditory Evoked Slow Waves Predict Post‐Sleep Cardiac Function
Source: J Sleep Res. 2026 Jun 1;35(5):e70354. doi: 10.1111/jsr.70354 (PMC13619023; doi:10.1111/jsr.70354)
Supplement: Supplementary file 1 [file JSR-35-e70354-s001.docx]

**Supplemental Materials:**

**Cardiovascular responses to natural and auditory evoked slow waves predict post-sleep cardiac function**

FIGURES

Fig S1

**Assessed for eligibility (n=38)**

**Excluded (n=12)**

♦  Not meeting inclusion criteria (n=5)

♦  Non-availability (n=7)

♦  Declined to participate (n=2)

**Excluded (n=7)**

♦  Not meeting inclusion criteria (n=7)

- sleep efficiency <70%: (n=3)
- signs of sleep apnea (AHI>: (n=2)
- high resting blood pressure: (n=2)

♦  Declined to participate (n=2)

**Entered main study (n=19)** 26) )

**Allocated to intervention (n=19)**

♦ Received allocated intervention and sham in a counter-balanced, crossed over design (n=18)

♦ Did not receive allocated intervention (n=1)

🡪 Exclusion due to non-adherence to study protocol (n=1)

**Analysed (n=17)**
♦ Exclusion of n = 1 due to technical failure in EEG recording of SHAM night

## Analysis

## Allocation

**Signed informed consent**

**Screening night (n=26)**

**CONSORT Flow Diagram for trial overview**

**Fig S2**

**Visual representation of type I and II slow waves.** Figures A and B provide a visual representation of slow wave types during an unstimulated night. The black signal shows the single-channel EEG derived from a multi-channel recording (in gray) after undergoing the pre-processing steps detailed in the methodology. The vertical dotted lines indicate the start and end of each slow wave. **(A)** illustrates a type I slow wave, while **(B)** shows a type II slow wave, both recorded from a 30-year-old male subject.

**Fig S3**

Effects of auditory stimulation on systolic blood pressure (BP) in the morning. Data is presented as mean ± standard error of the mean for n = 18 participants. P-values have been computed based on a linear-mixed effect model with the fixed factor Condition (*ISI1*_High_, *ISI1*_Low_, or SHAM) and random factor subject.

Fig S4

Effects of auditory stimulation on echocardiography parameter estimating right-ventricular systolic function. Right-ventricular tricuspid annular plane systolic excursion (TAPSE) is presented as mean ± standard error of the mean for n = 18 participants. P-values have been computed based on a linear-mixed effect model with the fixed factor Condition (*ISI1*_High_, *ISI1*_Low_, or SHAM) and random factor subject. Post-hoc comparison between conditions has been computed through paired t-tests and corrected for multiple comparisons by applying the Bonferroni correction. *: p < 0.05

**Figure S5**

Panels A–B show the waveforms of representative slow waves for Type I (A) and Type II (B), with the bold line indicating the average across electrodes. Panels C–E illustrate the corresponding scalp topographies: (C) Type I, (D) Type II, and (E) the relative difference. Both subtypes exhibit a frontal maximum, with Type I displaying stronger and more widespread activity, consistent with previous reports.

**Figure S6**

Cardiovascular responses to type I and type II slow waves during the ISI_Low_ condition. Event-related changes in instantaneous heart rate (IHR), systolic blood pressure (SBP), and diastolic blood pressure (DBP) are shown time-locked to the occurrence of Type I (blue) and Type II (orange) slow waves. Shaded areas represent the standard error of the mean (SEM). As in the SHAM condition (Fig. 4, main text), Type I slow waves are associated with larger transient cardiovascular responses than Type II waves. Statistical comparisons were conducted using the same within-subject tests as in the main figure (paired-samples t-tests across participants for each metric).

**Figure S7**

Cardiovascular responses to Type I and Type II slow waves during the ISI_High_ condition. Event-related changes in IHR, SBP, and DBP time-locked to Type I (blue) and Type II (orange) slow waves. The overall pattern replicates the SHAM and ISI_Low_ conditions, with Type I slow waves eliciting stronger cardiovascular responses than Type II. Shaded areas denote SEM. Statistical analysis followed the same procedure as in the main figure, confirming the robustness of the observed differences.

Figure S*6*: Cardiovascular responses to type I and type II slow waves during the ISI_Low_ condition. Event-related changes in instantaneous heart rate (IHR), systolic blood pressure (SBP), and diastolic blood pressure (DBP) are shown time-locked to the occurrence of Type I (blue) and Type II (orange) slow waves. Shaded areas represent the standard error of the mean (SEM). As in the SHAM condition (Fig. 4, main text), Type I slow waves are associated with larger transient cardiovascular responses than Type II waves. Statistical comparisons were conducted using the same within-subject tests as in the main figure (paired-samples t-tests across participants for each metric).

TABLES

Table S1: Included number of slow waves (mean +-SEM)

| Stimulated Slow waves Type I + II | **Sham** | **ISI_High_** | **ISI_low_** | **Lmer factor Condition** | **Posthoc** |
| --- | --- | --- | --- | --- | --- |
| **IHR** | 1010 ± 76.27 | 1041 ± 75.47 | 1064 ± 97.99 | F(2,28)=3.051  p=0.0633  N=15 | *NA* |
| **DPB** | 922.5 ± 70.95 | 947.8 ± 84.09 | 919.1 ± 100.8 | F(2,32)=0.073  p=0.9298  N=17 | *NA* |
| **SBP** | 922.5 ± 70.95 | 947.8 ± 84.09 | 919.1 ± 100.8 | F(2,32)=0.073  p=0.9298  N=17 | *NA* |

Table S2: Included number of Type I and Type II slow waves (mean +-SEM)

| Slow waves in SHAM | **Type I** | **Type II** | **Paired t-test** |
| --- | --- | --- | --- |
| **IHR** | 205.2 ± 90.7 | 2983.0 ± 1082.0 | T(16)= -11.31,  p = 4.9×10⁻⁹ |
| **DPB** | 196.4 ± 71.1 | 2871.0 ± 804.0 | T(16)= -14.53,  p = 1.2×10⁻¹⁰ |
| **SBP** | 196.4 ± 71.1 | 2871.0 ± 804.0 | T(16)= -14.53,  p = 1.2×10⁻¹⁰ |
